# Meta-analysis of the efficacy and impact on cardiac function of sodium–glucose cotransporter 2 inhibitor Empagliflozin in heart failure patients

**DOI:** 10.1097/MD.0000000000040409

**Published:** 2024-11-08

**Authors:** Weidong Li, Xuanyang Shen, Meiqi Zhang, Wentao Tan, Xiaolu Jiang, Hongfu Wen, Yuan Shen

**Affiliations:** a Department of Emergency, Affiliated Hospital of North Sichuan Medical College, Nanchong, China.

**Keywords:** cardiac function, clinical efficacy, Empagliflozin, heart failure, meta-analysis, sodium–glucose cotransporter 2 inhibitor

## Abstract

**Background:**

Currently, there is no comprehensive systematic review available to comprehensively assess the efficacy and safety of Empagliflozin and other sodium–glucose cotransporter 2 inhibitors in the treatment of heart failure (HF). This study employed a meta-analysis approach to systematically evaluate the therapeutic effects of Empagliflozin in HF patients and its impact on cardiac function.

**Method:**

The keywords including “heart failure,” “HF,” “cardiac failure,” “cardiac disease,” “Empagliflozin,” and “sodium–glucose cotransporter 2 inhibitors” were utilized to search for relevant clinical studies on Empagliflozin in the treatment of HF in various databases, such as China National Knowledge Infrastructure, Wanfang, VIP Chinese Medical Journal Database, PubMed, MEDLINE, Embase, Cochrane Library, Springer, and Science Direct. The studies included patients with HF who received drug treatment. Data on baseline characteristics and posttreatment outcomes, including HF hospitalization (HHF), cardiovascular mortality, all-cause mortality, estimated glomerular filtration rate changes, Kansas City Cardiomyopathy Questionnaire quality of life (QoL) scores, N-terminal pro-B-type natriuretic peptide, left ventricular ejection fraction, hematocrit, and other relevant indicators were collected. Meta-analysis was conducted using RevMan5.3 to analyze the extracted data.

**Results:**

A total of 15 studies were included in the final analysis, comprising 36,917 patients with HF. Among them, 18,486 patients were in Empagliflozin group, and 18,431 patients were in control (Ctrl) group. The results of the meta-analysis demonstrated that, relative to Ctrl group, Empagliflozin group showed a substantially lower HHF rate, a substantial improvement in estimated glomerular filtration rate changes, a reduced cardiovascular mortality rate, a higher Kansas City Cardiomyopathy Questionnaire QoL score, increased hematocrit values, reduced N-terminal pro-B-type natriuretic peptide changes, and enhanced left ventricular ejection fraction changes. These findings suggest that remarkable improvements in various outcomes compared to the Ctrl group.

**Conclusion:**

The sodium–glucose cotransporter 2 inhibitor Empagliflozin markedly reduces the HHF rate and cardiovascular mortality in HF patients. It also improves patients’ QoL, enhances renal function, and increases cardiac function while reducing both, the preload and afterload.

## 1. Introduction

Heart failure (HF) is a common and severe cardiovascular disorder that has attracted considerable global attention due to its high incidence and substantial disability rates.^[1]^ HF ranks as one of the leading causes of death worldwide, with millions of new cases diagnosed annually, primarily affecting the elderly population, especially those aged 65 and older.^[2]^ HF exhibits a higher prevalence in males at a younger age, while females experience an increased risk after menopause.^[3]^ Risk factors for HF encompass various elements, including coronary artery disease, hypertension, diabetes, myocardial disease, valvular heart disease, medications, among others.^[4,5]^ HF results in the heart’s inability to effectively pump blood, leading to insufficient oxygen and nutrient supply to the body, causing patients to frequently experience fatigue, shortness of breath, and weakness. This condition also leads to the accumulation of extracellular fluid in the body, causing edema and pulmonary congestion, which escalates the risk of respiratory distress. Additionally, HF patients may experience arrhythmias, increasing their susceptibility to stroke.^[6]^ HF detrimentally affects renal function, leading to declining kidney function and fluid retention in patients.^[7]^ As the disease progresses, HF causes damage to multiple organs and systems throughout the body, resulting in significant harm to the organism.^[8]^ Hence, effective preventative measures and treatment modalities play a crucial role in addressing HF.

HF is a complex disease, and the choice of treatment depends on the etiology, severity of symptoms, individual patient characteristics, and available resources. Currently, clinical management involves various approaches such as medication, implantable devices, surgical interventions, and lifestyle modifications. Among these, pharmacotherapy is a commonly employed treatment modality in clinical practice, effectively alleviating symptoms, reducing hospitalization rates, and enhancing patients’ quality of life (QoL). According to the Guidelines described in the “2021 ESC Guidelines for the diagnosis and treatment of acute and chronic heart failure,” commonly used medications in clinical treatment include: beta-blockers, RAAS inhibitors (angiotensin converting enzyme inhibitors and angiotensin receptor/neprilysin inhibitor), mineralocorticoid receptor antagonists and SGLT2i. In addition, diuretics are used to treat HF with congestion. Although these drugs provide some relief from clinical symptoms, a substantial portion of patients does not achieve satisfactory therapeutic outcomes.^[9]^ In recent years, novel treatment approaches have garnered the attention of researchers, notably SGLT2i Empagliflozin, an oral medication, is a widely utilized SGLT2i. It acts on a protein in the renal tubules responsible for glucose reabsorption, reducing blood glucose levels by decreasing glucose reabsorption in the renal tubules. As a result, it has found extensive application in the management of diabetes.^[10–13]^ Some researchers have pointed out that large-scale studies have shown that Empagliflozin can greatly reduce cardiovascular death, worsening of HF, and the risk of hospitalization in patients with type 2 diabetes. In recent years, several studies have explored the use of Empagliflozin in the treatment of cardiovascular diseases. Empagliflozin not only lowers the risk of hospitalization in HF patients but also alleviates HF symptoms and enhances cardiac function.^[14–16]^ These effects may be associated with its ability to reduce both cardiac preload and afterload, improve myocardial energy metabolism, and decrease renal tubular reabsorption of glucose.^[17,18]^

Despite some promising individual studies, there has been no systematic and comprehensive research to date that assesses the efficacy and safety of Empagliflozin and other SGLT2i in the treatment of HF. In this context, our study aimed to conduct a meta-analysis to summarize existing clinical research data and systematically evaluate the therapeutic effects of Empagliflozin in HF patients, as well as its impact on cardiac function. By pooling the results of various studies, this work aimed to gain a comprehensive understanding of the potential role of the SGLT2i Empagliflozin in HF management, providing a more robust scientific basis for decision-making and offering valuable clinical insights for the treatment and prognosis of HF.

## 2. Materials and methodologies

### 2.1. Literature retrieval methodologies

To retrieve relevant literature for our study, searches were conducted in various databases, including China National Knowledge Infrastructure, WanFang, VIP, PubMed, MEDLINE, Embase, Cochrane Library, Springer, Science Direct, and others. The search keywords comprised terms such as “heart failure,” “HF,” “cardiac failure,” “cardiac disease,” “Empagliflozin,” “SGLT2i,” and their variations. These keywords were combined into search strings, using Boolean operators (AND, OR, NOT) and parentheses when needed to ensure the accuracy of the search results. For example, the search string might be: (“HF” OR “cardiac failure” OR “cardiac disease”) AND (“Empagliflozin” OR “SGLT2i”). The search was conducted in each of the selected databases using the constructed search strings. This process was repeated across various databases to maximize the retrieval of relevant literature. The search period extended from the inception of the databases to July 2023. In addition to database searches, manual searches were performed, including reviewing journals in the relevant field, conference abstracts, and seeking expert opinions. Tracking citations in the references of included articles also lead to the discovery of more relevant studies. To organize, manage, and remove duplicates from the retrieved literature, we use reference management software like EndNote. This help streamline the search process and enhance efficiency.

### 2.2. Literature inclusion and exclusion criteria

The inclusion and exclusion criteria of retrieval documents are illustrated in Table 1.

**Table 1:** Literature inclusion and exclusion criteria.

Inclusion criteria	Exclusion criteria
1. The included literature must focus on patients with HF. This encompasses various types of HF, such as systolic HF, diastolic HF, and others. The study population should be explicitly identified as HF patients, as opposed to patients with other conditions.	1. Literature that was not relevant to HF patients, including studies related to other diseases or nonhuman studies.
2. The included literature must involve treatment interventions with Empagliflozin, with an emphasis on Empagliflozin as a standalone treatment.	2. Literature that did not provide data related to treatment outcomes, improvement in heart function, or data relevant to the research question. This includes review articles, brief reports, editorial comments, and similar types of publications.
3. The included literature must contain data related to treatment outcomes, improvement in heart function, or data relevant to the research question. This can include clinical trials, cohort studies, case-control studies, or other study types.	3. Literature that did not contain information about Empagliflozin treatment or where the study’s treatment intervention is not explicitly mentioned.
4. The literature should be in English or another language that is understandable to researchers.	4. Literature that was not in English or another language that can be understood by researchers.

### 2.3. Literature screening and quality evaluation methodologies

Literature selection was as follows. Following the literature search, an initial screening process was conducted. This involved assessing the literature based on the inclusion criteria, with a focus on reviewing the titles and abstracts to determine their potential relevance to the research objectives. Literature that appeared unrelated to the research questions was excluded at this stage. Subsequently, a full-text review was conducted for the remaining literature, examining complete research reports, including the methodology, results, and discussion sections. In cases where the literature did not provide sufficient information to determine its alignment with the criteria, attempts were made to contact the authors for additional details to further establish eligibility. Data relevant to the study, including study design, sample size, treatment interventions, and outcome data, were extracted from the literature that met the inclusion criteria. Duplicate literature was identified and removed using reference management software, such as EndNote, to ensure a refined selection of nonredundant sources.

Literature quality assessment was as follows. Appropriate quality assessment tools, often in the form of standardized assessment forms, were selected to evaluate the quality of each included study. In this case, since the included literature comprises randomized controlled trials, the Cochrane Collaboration’s risk of bias tool was employed for assessment. Each included study was evaluated for quality by 2 independent researchers. The assessors used predetermined criteria to assess the studies, which encompassed factors such as study design, risk of bias, data extraction, sample size, heterogeneity, and publication bias. Following the evaluation, a quality score was assigned to each study based on these assessments. This score was utilized for subsequent subgroup analysis or to weigh the inclusion studies. In the event of disagreements among assessors regarding the quality score of a particular study, the discrepancies were resolved through discussion and negotiation. When necessary, a willing third-party assessor was engaged to provide an additional perspective.

### 2.4. Data abstraction

Quality assessment in a meta-analysis is a crucial step, primarily evaluating aspects such as randomization, blinding, and sample size. Cochrane collaboration tools were primarily utilized for assessing the quality of included literature and other potential biases, including selection bias, recall bias, and detection bias. Sensitivity analyses can be performed to examine the impact of studies that did not meet the criteria on the analysis results. For instance, conducting a new meta-analysis after excluding lower-quality studies. Methods such as funnel plots were employed to assess the potential for publication bias.

Creating a structured Excel data extraction table is a vital component of the research process. This table should encompass a range of variables and information to be extracted, listing each variable, its definition, and the corresponding units, where applicable. More than 2 data extractors, all adequately trained, should be involved in the data extraction process to ensure both consistency and accuracy. These data extractors should meticulously review each full-text or detailed report of the included studies, following the guidance provided by the data extraction table. The primary information to be extracted should encompass the following:

A. *Basic information*: authors, publication data, study title, etc.
B. *Study design*: study type, sample size, randomization details, treatment interventions, duration of treatment, baseline data for variables such as heart rate, systolic blood pressure, left ventricular ejection fraction (LVEF), and N-terminal pro-B-type natriuretic peptide (NT-proBNP).
C. *Outcome data*: data for primary and secondary outcomes, including treatment efficacy, improvement in cardiac function, and adverse events; key statistical indicators, including HF hospitalization (HHF) rate, cardiovascular mortality rate, all-cause mortality rate, change in estimated glomerular filtration rate (eGFR) posttreatment, Kansas City Cardiomyopathy Questionnaire (KCCQ) QoL score posttreatment, NT-proBNP posttreatment, LVEF, and hematocrit.
D. *Statistical data*: sample sizes for each group, mean values, standard deviations, confidence intervals, etc.

### 2.5. Statistical methodologies

Meta-analysis of the included literature data was conducted using RevMan5.3. Effect sizes for each included study were computed, with quantitative data primarily represented as the mean difference (MD) and dichotomous data expressed as the odds ratio (OR). To visualize the effect sizes of individual studies and their pooled effects, a forest plot was generated to analyze the literature’s effect sizes. Heterogeneity analysis was carried out as follows: an initial assessment of literature heterogeneity was performed using a chi-squared test with a significance level set at α = 0.05 and a *P*-value of <.05. Subsequently, quantitative evaluation of heterogeneity results was conducted using the *I*^2^ statistic within RevMan5.3. If *I*^2^ was <50%, a fixed-effects model was applied for the meta-analysis. If *I*^2^ exceeded 50%, a random-effects model (REM) was utilized for the meta-analysis. Publication bias was assessed by creating a funnel plot to examine if there was any bias in the included literature. Statistical significance was defined as *P* < .05 to determine the presence of inter-group differences.

## 3. Results

### 3.1. Search results of literature

**Figure 1.**
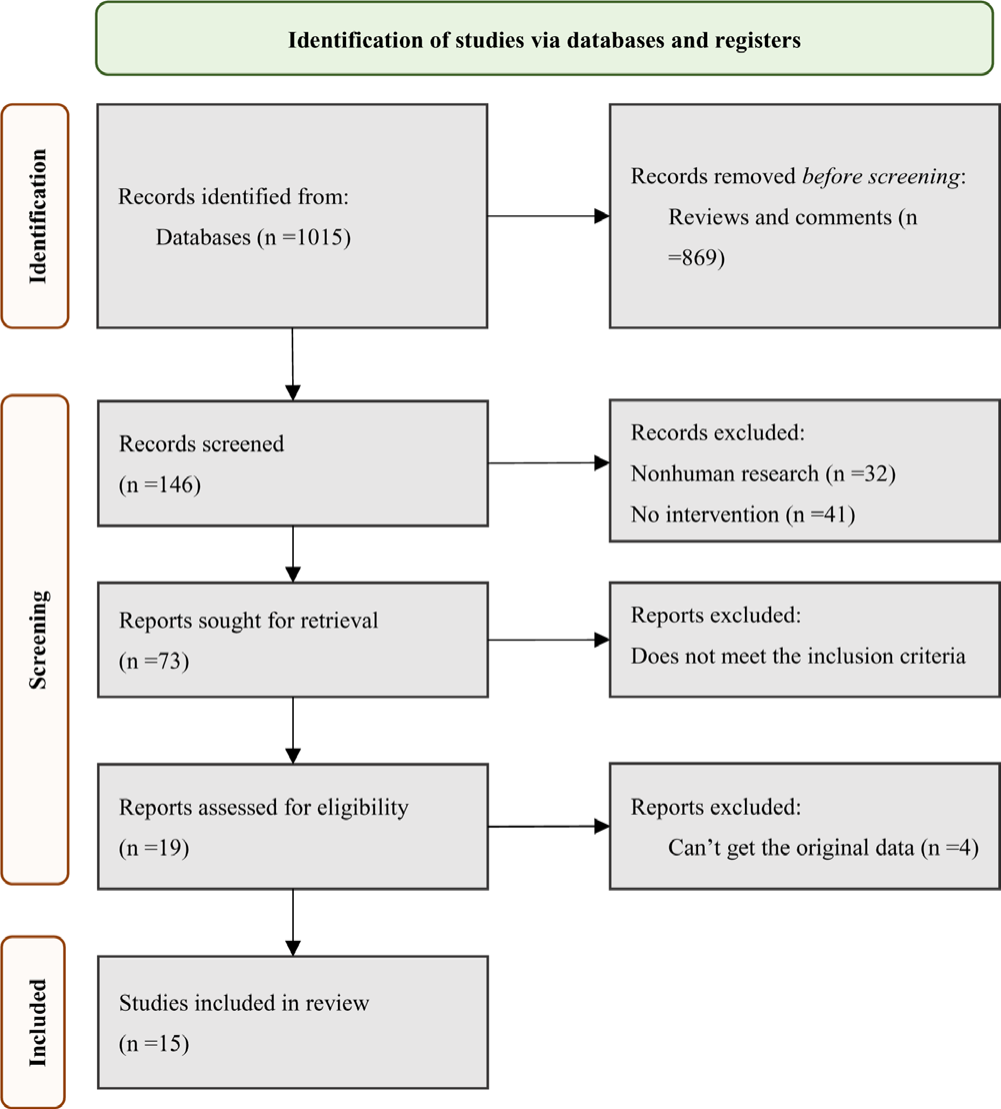
Basic process of document retrieval.

A total of 1015 relevant publications were identified through keyword searches in various databases. After the initial screening, which involved removing reviews and commentaries, 146 publications remained. Further exclusions were made for 32 nonhuman studies and 41 studies that did not pertain to therapeutic interventions. This left 73 publications for further review. After scrutinizing the titles and abstracts to ensure alignment with inclusion criteria, 54 publications were excluded. A comprehensive assessment of the remaining publications led to the removal of 4 studies due to the unavailability of original data. Ultimately, *I*^2^ publications met the criteria and were included in the analysis, focusing on the impact of SGLT2i Empagliflozin on the efficacy and cardiac function of HF patients. The literature search and screening process for this study are depicted in Figure 1.

### 3.2. Basic information included in the literature

A total of 15 publications^[19–33]^ were included in this analysis, comprising 36,917 patients with HF. Among these, 18,486 patients received Empagliflozin treatment, while 18,431 patients served as controls. Table 2 provides a detailed summary of the basic information from the included publications, including the first author, publication year, number of patients, age, gender, treatment methodologies, treatment duration, percentage of patients with New York Heart Association functional class II-IV prior to treatment, heart rate, systolic blood pressure, LVEF, and NT-proBNP levels.

**Table 2:** Basic data and baseline data of included literature.

Author	Year	Country	Study type	Total case	Case number		Therapeutic regimen		Treatment time (weeks)	Age (years old)	
					Intervention group	Placebo group	Intervention group	Placebo group		Intervention group	Placebo group
Anker SD^[1]^	2021	Germany	RCT	5988	2997	2991	10 mg Empagliflozin	10 mg placebo	112	71.8 ± 9.3	71.9 ± 9.6
Butler J^[2]^	2022	USA	RCT	5988	2997	2991	10 mg Empagliflozin	10 mg placebo	52	71.0 ± 9.4	73.0 ± 9.4
Ferreira JP^[3]^	2022	France	RCT	9718	4860	4858	10 mg Empagliflozin	10 mg placebo	208	69.9 ± 10.4	70.0 ± 11.0
Filippatos G^[4]^	2022	Greece	RCT	5988	2997	2991	10 mg Empagliflozin	10 mg placebo	52	70.9 ± 9.0	72.8 ± 9.7
HundertmarkMJ^[5]^	2023	UK	Prospective double-blind RCT	72	35	37	10 mg Empagliflozin	10 mg placebo	12	67.5 ± 12.7	64.7 ± 14.1
Jensen J^[6]^	2020	Denmark	Double-blind RCT	190	95	95	10 mg Empagliflozin	10 mg placebo	12	64 ± 11.9	63 ± 12.59
Lee MMY^[7]^	2021	UK	Double-blind RCT	105	52	53	10 mg Empagliflozin	10 mg placebo	26	64.3 ± 11.7	69.2 ± 10.6
Nassif ME^[8]^	2021	USA	Multicenter double-blind RCT	65	33	32	10 mg Empagliflozin	10 mg placebo	12	69.5 ± 12.0	62.9 ± 13.3
Omar M^[9]^	2021	Denmark	Double-blind RCT	190	95	95	10 mg Empagliflozin	10 mg placebo	12	65 ± 10	63 ± 12
Packer M^[10]^	2021	UK	Double-blind RCT	2249	1139	1110	10 mg Empagliflozin	10 mg placebo	12	66.4 ± 11.2	66.7 ± 10.8
Packer M^[11]^	2020	UK	RCT	3730	1863	1867	10 mg Empagliflozin	10 mg placebo	69	67.2 ± 10.8	66.5 ± 11.2
Requena-Ibáñez JA^[12]^	2021	USA	RCT	84	42	42	10 mg Empagliflozin	10 mg placebo	6	64.2 ± 10.9	59.9 ± 13.1
Santos-Gallego CG^[13]^	2021	USA	Double-blind RCT	84	42	42	10 mg Empagliflozin	10 mg placebo	6	64.2 ± 10.9	59.9 ± 13.1
Voors AA^[14]^	2022	Netherlands	RCT	530	265	265	10 mg Empagliflozin	10 mg placebo	12	71.0 ± 3.68	70 ± 4.37
Zannad F^[15]^	2021	UK	RCT	1746	879	867	10 mg Empagliflozin	10 mg placebo	112	63.7 ± 11.2	62.3 ± 11.3

Gender (female/male)		NYHA Class ≥ II		HR (beats/min)		SBP (mm Hg)		LVEF (%)		NT-proBNP (pg/mL)	
Intervention group	Placebo group	Intervention group	Placebo group	Intervention group	Placebo group	Intervention group	Placebo group	Intervention group	Placebo group	Intervention group	Placebo group
/	/	/	/	/	/	/	/	/	/	/	/
437/1426	456/1411	1399	1401	71.0 ± 11.7	71.5 ± 11.8	122.6 ± 15.9	121.4 ± 15.4	27.7 ± 6.0	27.2 ± 6.1	1887 (1077–3429)	1926 (1153–3525)
1338/1659	1338/1653	2432	2451	70.4 ± 12.0	70.3 ± 11.8	131.8 ± 15.6	131.9 ± 15.7	54.3 ± 8.8	54.3 ± 8.8	994 (501–1740	946 (498–1725)
86/179	93/172	95	91	/	/	120 ± 19.2	122 ± 20.7	31 ± 6.28	32 ± 1.97	3299 (1843–6130)	3106 (1588–6013)
282/857	280/830	243	221	70.3 ± 11.5	71.0 ± 11.3	122.2 ± 15.7	121.6 ± 15.5	27.8 ± 5.3	27.1 ± 5.9	1805 (1032–3191)	1772 (1074–3155)
204/675	183/684	683	671	71.9 ± 11.9	71.8 ± 12.1	122.4 ± 15.5	120.1 ± 14.9	27.4 ± 6.0	26.8 ± 6	1505 (907, 2511)	1548 (914, 2650)
28/77	18/34	37	44	68.0 ± 12.9	64.8 ± 9.8	125.8 ± 18.2	130.3 ± 21.6	/	/	/	/
15/20	15/22	27	32	72.6 ± 16.2	72.4 ± 10.7	123.5 ± 24.8	120.1 ± 16.1	36.9 ± 9.2	39.9 ± 9.8	801.1 ± 3.3	800.9 ± 2.5
/	/	/	/	/	/	/	/	/	/	/	/
12/21	12/20	14	16	70.1 ± 10.2	72.6 ± 9.8	128.3 ± 19.1	120.7 ± 20.3	/	/	140.0 (85.0, 286.0)	129.0 (51.0, 378.5)
15/27	15/27	/	/	74.0 ± 17.0	78.0 ± 15.0	/	/	/	/	/	/
15/27	15/27	/	/	72.0 ± 15.0	79.0 ± 15.0	/	/	/	/	/	/
16/79	12/79	72	77	68 ± 10.37	70 ± 12.59	117 ± 17.04	122 ± 17.04	30 ± 7.41	30 ± 7.41	582 (304–1020)	605 (322–1070)
16/79	12/83	72	77	69 ± 11	72 ± 13	119 ± 18	121 ± 16	/	/	582 (303–1020)	605 (309–1080)
/	/	/	/	/	/	/	/	/	/	/	/

HR = heart rate, LVEF = left ventricular ejection fraction, NT-proBNP = N-terminal pro-B-type natriuretic peptide, NYHA = New York Heart Association, RCT = randomized controlled trial, SBP = systolic blood pressure, UK = The United Kingdom of Great Britain and Northern Ireland, USA = The United States of America.

### 3.3. Quality evaluation of included literature

**Figure 2.**
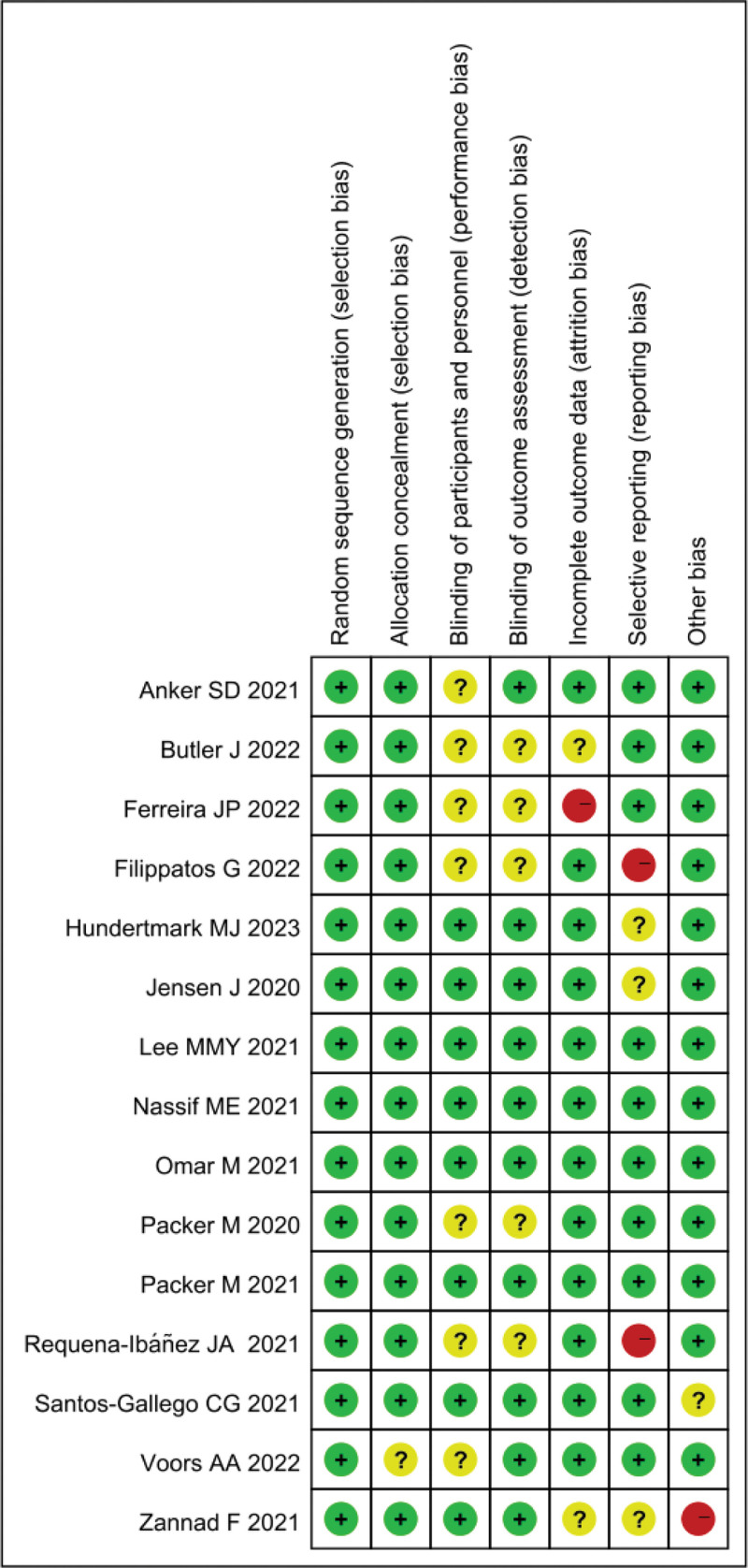
Summary of risk assessment for inclusion in literature bias.

The quality of the 15 included publications was assessed using the Cochrane Reviewer’s Handbook. A comprehensive risk of bias assessment graph was created using RevMan5.3. One publication had a “high risk” in this domain, 2 publications were rated as “high risk” in this category. Additionally, there were 2 publications with “unclear risk” and “high risk” for other biases, respectively (Fig. 2).

### 3.4. The effect of Empagliflozin on HHF rate

**Figure 3.**
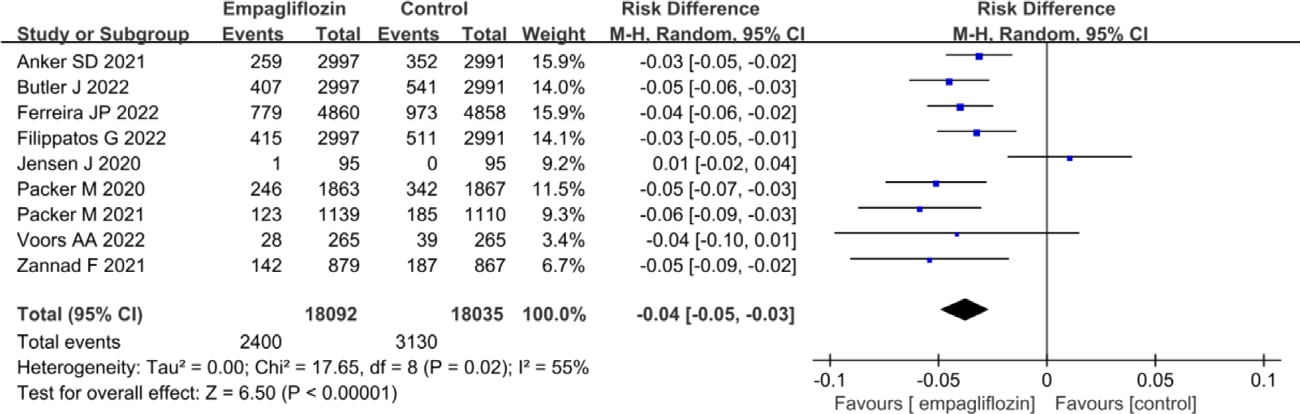
Forest plot of the impact of Empagliflozin on HHF rate. HHF = heart failure hospitalization.

The statistical results of HHF rates for Empagliflozin group and control (Ctrl) group are illustrated in Figure 3. Marked heterogeneity existed in the HHF rates between Empagliflozin group and Ctrl group (*I*^2^ = 50%, *P* = .02). Hence, a REM was employed for the analysis. The results revealed that the HHF rate in HF patients after treatment with Empagliflozin was markedly inferior to that in Ctrl group (OR = ‐0.04, 95% CI: −0.05 to −0.03; *Z* = 6.50, *P* < .00001).

### 3.5. The effect of Empagliflozin on mortality rate

**Figure 4.**
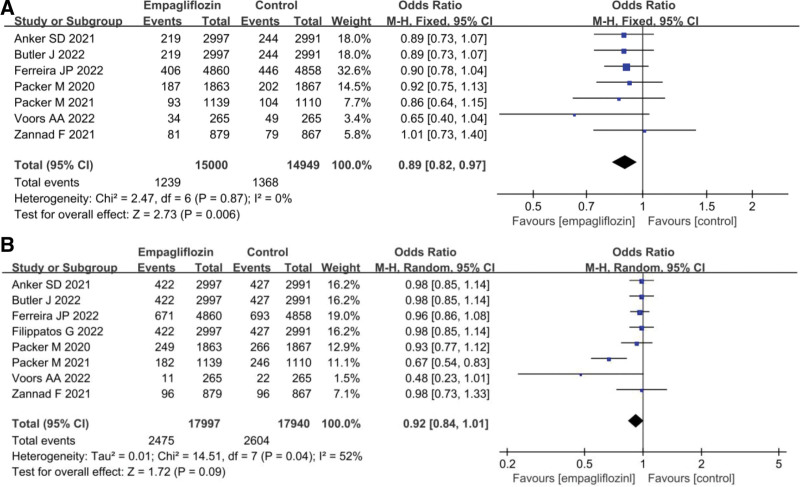
Forest map of the impact of Empagliflozin on mortality rate. (A) Cardiovascular disease mortality rate; (B) All-cause mortality rate.

The statistical results of cardiovascular disease mortality and all-cause mortality rates for Empagliflozin group and Ctrl group are presented in Figure 4. No considerable heterogeneity was indicated in the cardiovascular disease mortality rates between Empagliflozin group and Ctrl group (*I*^2^ = 0%, *P* = .87); therefore, a fixed-effects model was utilized for the analysis. In the case of all-cause mortality rates, there was heterogeneity (*I*^2^ = 52%, *P* = .04), and a REM was applied for the analysis. The results indicated that Empagliflozin group exhibited a lower cardiovascular disease mortality rate versus Ctrl group (OR = 0.89, 95% CI: 0.82 to 0.97, *Z* = 2.73, *P* = .006), while neglectable difference existed in the all-cause mortality rate versus Ctrl group (OR = 0.92, 95% CI: 0.84 to 1.01, *Z* = 1.72, *P* = .09).

### 3.6. KCCQ QoL score after treatment

**Figure 5.**
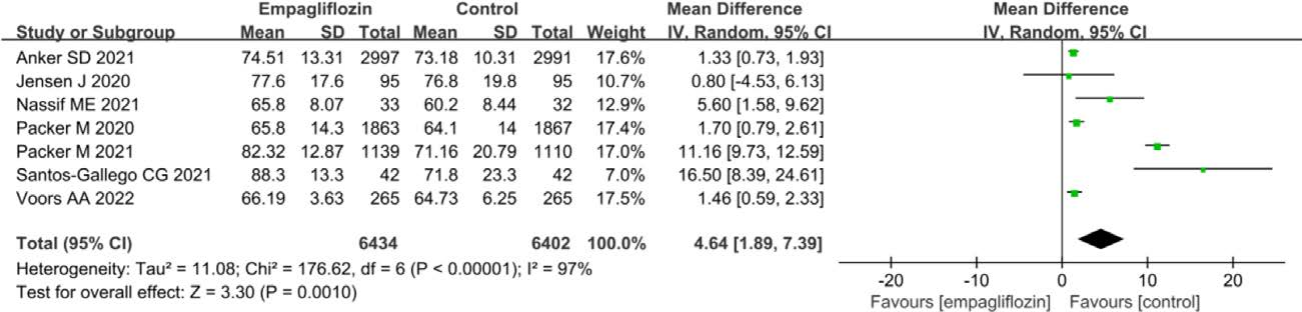
Forest map of the effect of Empagliflozin on KCCQ QoL score. KCCQ QoL = Kansas City Cardiomyopathy Questionnaire quality of life.

The statistical results of the KCCQ QoL scores after treatment for Empagliflozin group and Ctrl group are presented in Figure 5. Notable heterogeneity was observed between the KCCQ QoL scores of Empagliflozin group and Ctrl group (*I*^2^ = 97%, *P* < .0001). Hence, a REM was utilized for the analysis. The results indicated that Empagliflozin group exhibited drastically superior KCCQ QoL scores after treatment versus Ctrl group (MD = 4.64, 95% CI: 1.89 to 7.39, *Z* = 3.30, *P* = .001).

### 3.7. Changes in eGFR before and after treatment

**Figure 6.**
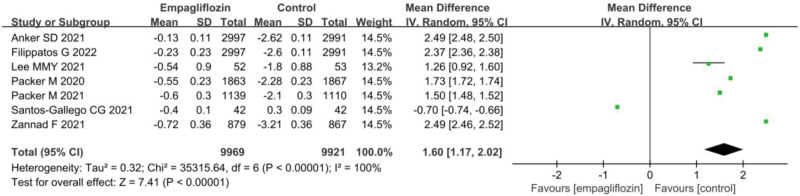
Forest plot of the impact of Empagliflozin on the changes in eGFR before and after treatment. eGFR = estimated glomerular filtration rate.

The statistical results for the eGFR change values before and after treatment for Empagliflozin group and Ctrl group are presented in Figure 6. Considerable heterogeneity was observed between the eGFR change values before and after treatment in Empagliflozin group and Ctrl group (*I*^2^ = 100%, *P* < .0001). Hence, a REM was utilized for the analysis. The results indicated that Empagliflozin group exhibited a markedly inferior eGFR change value before and after treatment to Ctrl group (MD = ‐2.49, 95% CI: −1.89 to −7.39, *Z* = 3.30, *P* = .001).

### 3.8. Comparison of hematocrit values after treatment

**Figure 7.**
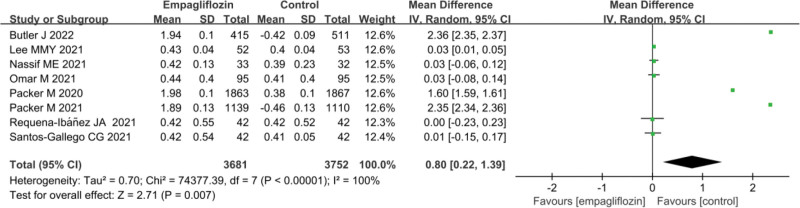
Forest map of the effect of Empagliflozin on the hematocrit value after treatment.

The statistical results for the hematocrit values after treatment in Empagliflozin group and Ctrl group are presented in Figure 7. There was substantial heterogeneity observed between the hematocrit values after treatment in Empagliflozin group and Ctrl group (*I*^2^ = 100%, *P* < .0001). Hence, a REM was utilized for the analysis. The results indicated that Empagliflozin group exhibited a drastically superior hematocrit value after treatment to Ctrl group (MD = 0.80, 95% CI: 0.22 to 1.39, *Z* = 2.71, *P* = .007).

### 3.9. Changes in cardiac function indicators after treatment

**Figure 8.**
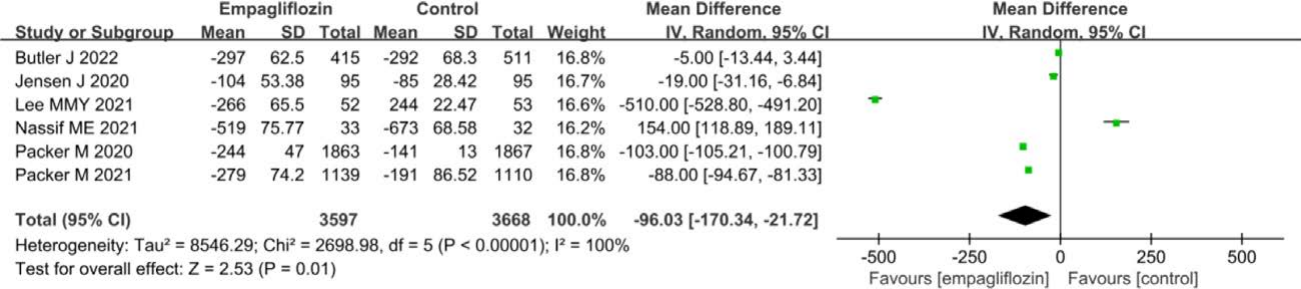
Forest plot of the effect of Empagliflozin on the changes in NT-proBNP before and after treatment. NT-proBNP = N-terminal pro-B-type natriuretic peptide.

The statistical results for the change in NT-proBNP values before and after treatment in Empagliflozin group and Ctrl group are presented in Figure 8. Considerable heterogeneity was observed between the change in NT-proBNP values before and after treatment in Empagliflozin group and Ctrl group (*I*^2^ = 100%, *P* < .0001). Hence, a REM was utilized for the analysis. The results indicated that Empagliflozin group exhibited a greatly superior change in NT-proBNP values before and after treatment versus Ctrl group (MD = ‐96.03, 95% CI: −170.34 to −21.72, *Z* = 2.53, *P* = .01).

**Figure 9.**
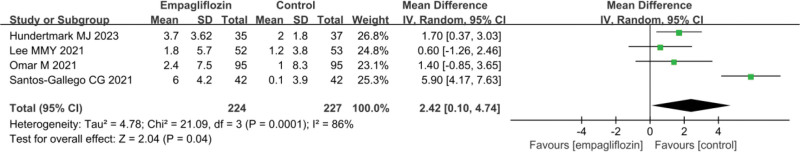
Forest plot of the effect of Empagliflozin on changes in LVEF before and after treatment. LVEF = left ventricular ejection fraction.

The statistical results for the change in LVEF values before and after treatment in Empagliflozin group and Ctrl group are presented in Figure 9. Notable heterogeneity existed between the change in LVEF values before and after treatment in Empagliflozin group and Ctrl group (*I*^2^ = 86%, *P* = .0001). Hence, a REM was utilized for the analysis. The results indicated that the change in LVEF values before and after treatment in Empagliflozin group was higher than that in Ctrl group, and substantial difference was found in the change in LVEF values before and after treatment between the 2 groups (MD = 2.42, 95% CI: 0.10 to 4.74, *Z* = 2.04, *P* = .04).

### 3.10. Publication bias analysis

**Figure 10.**
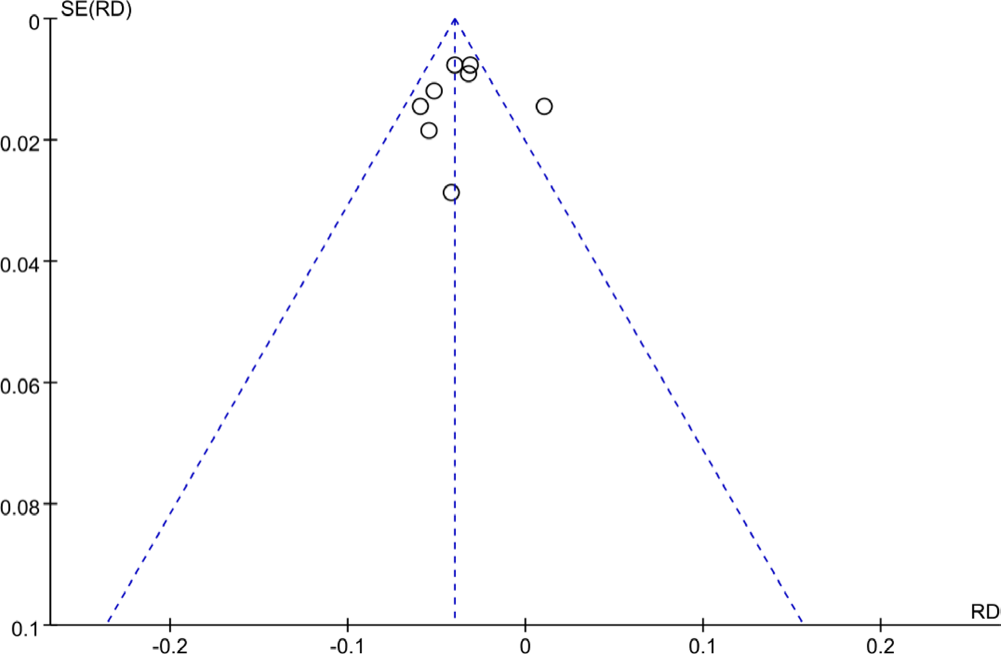
Funnel plot of the effect of Empagliflozin on hospitalization rate for HF (HHF). HHF = heart failure hospitalization.

**Figure 11.**
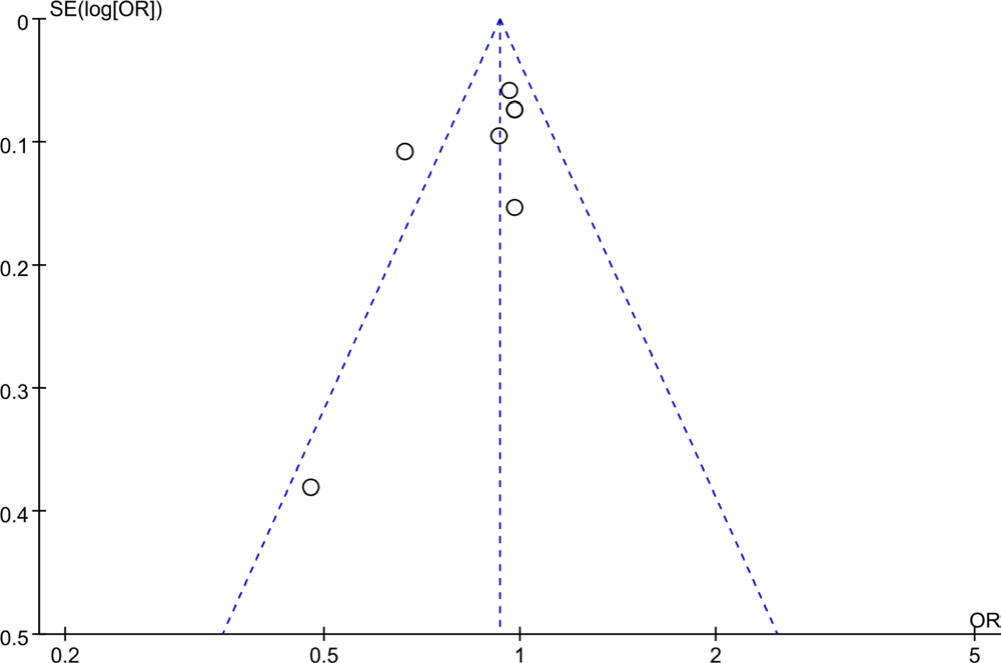
Funnel plot of the impact of Empagliflozin on all-cause mortality rate.

A funnel plot was employed to analyze publication bias in the included literature of this study. The results, as depicted in Figures 10 and 11, demonstrated that the funnel plot of the included literature in this study exhibited a symmetrical distribution of data points, indicating the absence of significant publication bias.

## 4. Discussion

Empagliflozin is a drug classified as a SGLT2i, which is a protein located in the renal tubules responsible for reabsorbing glucose back into the bloodstream during the process of urine reabsorption.^[34]^ Empagliflozin works by inhibiting SGLT2, reducing the reabsorption of glucose by renal tubules, thereby increasing the excretion of glucose in the urine and is primarily utilized for diabetes treatment.^[35]^ In recent years, it has also been studied and utilized in the treatment of HF. Empagliflozin has been shown to significantly reduce the risk of hospitalization in clinical trials involving HF patients,^[36]^ making it a promising drug for the treatment of chronic HF. Furthermore, research indicates that Empagliflozin can improve the QoL for HF patients, alleviate symptoms, enhance exercise capacity, and reduce the mortality rate among HF patients.^[37]^ Some studies suggest that in certain patient groups, such as those with HF accompanied by diabetes, Empagliflozin may be particularly effective.^[38,39]^ Nevertheless, not all HF patients experience the same benefits from this medication, and therefore, there is still ongoing debate regarding the effectiveness of Empagliflozin in the treatment of HF. The study utilized a meta-analysis approach to assess the efficacy of Empagliflozin in treating HF. The results revealed that, in comparison to Ctrl group, Empagliflozin significantly reduces the risk of HHF (OR = ‐0.04, 95% CI: −0.05 to −0.03; *Z* = 6.50, *P* < .00001). This analysis suggests that these findings may be attributed to mechanisms such as Empagliflozin reducing fluid retention, decreasing cardiac load, and improving cardiac metabolism. Empagliflozin achieves this by promoting the excretion of additional glucose and sodium by the kidneys, leading to fluid elimination. This contributes to alleviating the problem of fluid retention in patients with HF, resulting in the reduction of symptoms like pulmonary congestion and weight gain, ultimately lowering the risk of hospitalization due to fluid retention.^[40]^ The DAPA-HF trial results also support these findings,^[41]^ indicating that HF patients treated with SGLT2i, including Empagliflozin, have a greatly reduced risk of HF-related hospitalization and HF events, underscoring the benefits of Empagliflozin in HF management, including reducing the risk of worsening HF and hospitalization.

Currently, multiple studies have indicated that HF patients treated with Empagliflozin experience a drastic reduction in cardiovascular mortality and all-cause mortality.^[19,32,42]^ The results of this meta-analysis demonstrated that the cardiovascular disease mortality rate in Empagliflozin group was inferior to that in Ctrl group (OR = 0.89, 95% CI: 0.82–0.97, *Z* = 2.73, *P* = .006). The all-cause mortality demonstrated neglectable difference versus Ctrl group (OR = 0.92, 95% CI: 0.84–1.01, Z = 1.72, *P* = .09). These findings suggest that Empagliflozin treatment greatly reduces cardiovascular disease mortality in HF patients, demonstrating a marked advantage in terms of cardiovascular disease mortality. Nevertheless, further research is needed to fully understand its impact on all-cause mortality. The KCCQ is a standard questionnaire utilized to assess the QoL in HF patients. It covers HF symptoms, limitations in daily activities, physical discomfort, cardiac symptoms, heart treatment, self-perception, psychological well-being, and social interactions, among other factors. A higher total score indicates milder HF symptoms and better QoL for patients.^[43]^ The results presented in this work indicated that Empagliflozin group’s posttreatment KCCQ QoL score was substantially superior to that of Ctrl group (MD = 4.64, 95% CI: 1.89–7.39, Z = 3.30, *P* = .001). This finding suggests that Empagliflozin treatment can markedly enhance the QoL for HF patients. This is a crucial discovery, as improving patients’ QoL is typically a vital goal in HF treatment. This improvement may be attributed to the fact that SGLT2i can alleviate HF symptoms, reduce hospitalization risks, enhance exercise tolerance, and make patients feel more energetic, leading to an overall improved QoL.

eGFR is an indicator utilized to estimate renal filtration function, serving as an assessment of kidney health. It is typically associated with the diagnosis and management of conditions such as chronic kidney disease and HF.^[44]^ Drugs like Empagliflozin are believed to have a certain protective effect on renal function.^[45]^ Hence, monitoring changes in eGFR is valuable in understanding treatment outcomes. Currently, multiple research findings indicate that patients treated with Empagliflozin experience a prominent reduction in eGFR. The results revealed that the change in eGFR values before and after treatment in Empagliflozin group was markedly inferior to in Ctrl group (MD = 2.49, 95% CI: 1.89–7.39, Z = 3.30, *P* = .001). This outcome suggests that under Empagliflozin treatment, eGFR greatly decreases in HF patients, indicating that Empagliflozin therapy has a discernible impact on renal function. Empagliflozin induces the excretion of excess glucose and sodium by the kidneys, leading to an increase in the excretion of glucose and sodium in urine and subsequently an increase in urine volume, potentially resulting in a decrease in eGFR.^[46]^ Furthermore, Empagliflozin leads to increased urinary excretion, which may reduce the body’s fluid volume. A decrease in bodily fluids can affect blood volume, thereby impacting the glomerular filtration rate of the kidneys.^[47]^ This might also explain the eventual decrease in eGFR.

Hematocrit value is an indicator utilized to assess blood composition, representing the proportion of red blood cells in the total blood volume. In patients with HF, the diminished pumping capacity of the heart may lead to fluid retention and congestion, which can affect the degree of blood dilution.^[48]^ An increase in hematocrit value may suggest a decrease in blood volume, while a decrease in hematocrit value may imply excessive fluid retention. Some HF patients may experience anemia, characterized by a lower-than-normal count of red blood cells or hemoglobin levels, which often results in a decrease in hematocrit value.^[49]^ Anemia can lead to symptoms such as fatigue, shortness of breath, and weakness, making changes in hematocrit value a useful indicator to evaluate a patient’s anemic status.^[50]^ The results indicated that hematocrit values in Empagliflozin group were considerably superior to in Ctrl group after treatment (MD = 0.80, 95% CI: 0.22–1.39, *Z* = 2.71, *P* = .007). This suggests that the use of Empagliflozin treatment leads to a significant increase in patients’ hematocrit values. An increase in hematocrit value typically signifies an elevation in the proportion of red blood cells in the blood, resulting in more concentrated blood. This phenomenon may be related to the treatment mechanism of Empagliflozin, including its impact on the kidneys, leading to fluid excretion and sodium loss, ultimately causing the blood to become more concentrated.^[51]^

NT-proBNP is a biomarker that doctors can use to determine the presence of HF when patients exhibit HF-related symptoms such as shortness of breath and edema. Elevated levels of NT-proBNP typically indicate the possibility of HF.^[52]^ Furthermore, NT-proBNP levels are often associated with the severity of HF, with higher NT-proBNP levels associated with lower LVEF and higher hospitalization rates.^[53]^ Lastly, NT-proBNP levels can be employed to monitor the effectiveness of HF treatment. Following treatment, a decrease in NT-proBNP levels generally indicates treatment efficacy and an improvement in HF symptoms.^[54]^ The results presented in this work indicated that the change in NT-proBNP levels before and after treatment in Empagliflozin group was notably superior to in Ctrl group (MD = ‐96.03, 95% CI: −170.34 to ‐21.72, *Z* = 2.53, *P* = .01). This suggests that posttreatment, patients with HF who received Empagliflozin experienced a more notable decrease in NT-proBNP levels, indicating that Empagliflozin treatment can improve HF symptoms. LVEF is a critical indicator utilized to assess heart function. LVEF measures the percentage of blood pumped out by the left ventricle with each heartbeat. A higher LVEF value indicates better heart function, as it can pump out more blood. Conversely, a lower LVEF value signifies poorer heart function. In patients with HF, LVEF values are typically lower, indicating the heart’s inability to effectively pump blood, which is one of the key features of HF.^[55]^ Lower LVEF is often associated with a worse prognosis, including the worsening of HF, increased risk of hospitalization, and a higher risk of sudden cardiac death. The results revealed that the change in LVEF values before and after Empagliflozin treatment was superior to in Ctrl group (MD = 2.42, 95% CI: 0.10–4.74, *Z* = 2.04, *P* = .04). This suggests that after treatment with Empagliflozin, patients experienced a drastic increase in LVEF values, while patients in Ctrl group did not exhibit such a notable improvement. An increase in LVEF typically indicates improved heart function, meaning the heart is pumping more effectively. The elevation of LVEF is usually associated with improved cardiac function and symptom relief.

### 4.1. Limitations

This study has certain limitations. First, the sample size is relatively small, which could lead to reduced sensitivity in detecting some effects and potential selection bias. Second, the study did not consider potential differences in the response to Empagliflozin treatment among different HF subtypes, such as HFpEF, HFrEF, and HFmrEF. Future research can expand the sample size, extend the study duration, and thoroughly investigate differences among various HF subtypes to further explore the effectiveness of Empagliflozin treatment in HF patients. Additionally, further research can focus on the long-term effects of Empagliflozin treatment, including its impact on patient prognosis and survival rates.

## 5. Conclusion

This study, based on a meta-analysis approach, explored the effects of the SGLT2i Empagliflozin on HF patients and their cardiac function. The results indicated that Empagliflozin, an SGLT2i, greatly reduces the HF patients’ risk of HHF and cardiovascular mortality, improves their QoL, enhances renal and cardiac function, and plays a positive role in HF treatment. In summary, the results of this study provide preliminary evidence for the application of Empagliflozin in the treatment of HF.

## Author contributions

**Conceptualization:** Weidong Li.

**Data curation:** Weidong Li, Xuanyang Shen, Meiqi Zhang.

**Formal analysis:** Weidong Li, Xuanyang Shen, Meiqi Zhang, Wentao Tan, Hongfu Wen, Yuan Shen.

**Investigation:** Weidong Li, Xuanyang Shen, Meiqi Zhang, Wentao Tan, Hongfu Wen.

**Methodology:** Xuanyang Shen, Xiaolu Jiang.

**Software:** Xuanyang Shen, Meiqi Zhang, Wentao Tan, Xiaolu Jiang, Hongfu Wen, Yuan Shen.

**Supervision:** Wentao Tan.

**Validation:** Wentao Tan.

**Visualization:** Xuanyang Shen, Xiaolu Jiang, Yuan Shen.

**Writing – original draft:** Weidong Li, Xuanyang Shen, Meiqi Zhang.

**Writing – review & editing:** Weidong Li, Xuanyang Shen.
